# Whortleberry protects kidney against the cisplatin-induced nephrotoxicity: an experimental study

**DOI:** 10.1080/0886022X.2018.1500292

**Published:** 2018-08-21

**Authors:** Huseyin Eren, Hasan Riza Aydin, Levent Tumkaya, Ilke Onur Kazaz, Yildiray Kalkan, Seher Nazli Kazaz, Tolga Mercantepe, Mustafa Ozan Horsanali, Adnan Yilmaz

**Affiliations:** a Urology Department, Recep Tayyip Erdogan University School of Medicine, Rize, Turkey;; b Urology Department, Kanuni Training and Research Hospital, Medical Science University School of Medicine, Trabzon, Turkey;; c Histology and Embryology Department, Recep Tayyip Erdogan University School of Medicine, Rize, Turkey;; d Urology Department, School of Medicine, Karadeniz Technical University, Trabzon, Turkey;; e Medical Oncology Department, Kanuni Training and Research Hospital, Trabzon, Turkey;; f Urology Department, Recep Tayyip Erdogan University Training and Research Hospital, Rize, Turkey;; g Biochemistry Department, Recep Tayyip Erdogan University School of Medicine, Rize, Turkey

**Keywords:** Cisplatin**;** nephrotoxicity, kidney, total antioxidant status, total oxidant status, whortleberry

## Abstract

**Purpose:** This study investigated the antioxidant effects of whortleberry against cisplatin-induced nephrotoxicity in rats.

**Material and methods:** This study included 48 female Sprague–Dawley rats weighing 263.68 ± 8.29 g. The rats were divided into the following six groups, with eight rats in each group: control, ethanol control, whortleberry control, cisplatin control, 16 mg/kg cisplatin +100 mg/kg whortleberry, and 16 mg/kg cisplatin +200 mg/kg whortleberry groups. Biochemical analysis was performed by measuring total oxidant status and total antioxidant status, histopathological analysis was performed by calculating proximal and distal tubule areas (μm^2^), and immunohistochemical analysis was performed by determining anti-Caspase-3 immunostaining. Differences among the groups were examined using one-way analysis of variance, and *p* < .05 was considered statistically significant.

**Results:** Cisplatin treatment decreased the total antioxidant status and increased the total oxidant status and Caspase-3 level. Moreover, it resulted in the dilatation, vacuolization and loss of tubular epithelial cells; and glomerular degeneration and edema in the kidney tissues (*p* < .05). Treatment with 100 and 200 mg whortleberries increased the total antioxidant status; decreased the total oxidant status and Caspase-3 level and ameliorated distal and proximal tubule degeneration, glomerular degeneration and edema in the kidney tissues (*p* < .05).

**Conclusions:** Our results indicate that the antioxidant effects of the whortleberry decrease cisplatin-associated nephrotoxicity.

## Introduction

Cisplatin is a chemotherapeutic agent used against solid tumors such as small cell-lung, obstetric, urogenital, breast, and neck cancer [1,2]. Not only it has significant anticancer activity but also it has side effects like nephrotoxicity, ototoxicity, neurotoxicity, and hepatotoxicity and these side effects could limit the using of it [3].

As we know that cisplatin generally accumulates in the proximal tubule cells and damages it by the via cellular necrosis, a decrease of microvillus, difference of lysosomes, and mitochondrial vacuolization [4]. At the same time, cisplatin causes lipid peroxidation and changes the amount of glutathione which results in nephrotoxicity in the kidney [5]. Although it is not still known how cisplatin causes the nephrotoxicity, it is blamed that its nephrotoxicity associated with free radical-mediated oxidative damage, reducing glutathione levels, and macromolecule synthesis in kidneys [6].

Nowadays, nutritionists are studying on avoiding harmful effects of oxygen molecules which impair oxidation processes of human bodies [7]. Different studies have demonstrated that antioxidants could prevent free radicals formation or reduce the producing of free radicals by disrupting oxidation chain reactions [8–10]. Antioxidants impair free radicals formations and lipid peroxidation. As a result, reducing lipid peroxidation via impairs the generation of free radicals and reduces damages induced by enzymes [11]. It was demonstrated that all bilberries (*Vaccinium myrtillus*) and one of its member whortleberry *(V. myrtillus L.)* have the high antioxidative capacity, decrease low-density lipoprotein oxidation, and vasoprotective anti-inflammatory activity [12,13]. In addition, whortleberry reduces nitric oxide (NO) levels as well [13]. Bilberry’s antioxidant effects are associated with its anthocyanin contents [14]. Anthocyanin reduces free radicals, prevents NO formation, decreases oxidative stress, reduces malondialdehyde (MDA), decreases advanced oxidation protein products (AOPP), and stabilizes physiological functions in the normal range [15–18].

Bilberries like whortleberries are grown and consumed excessively in our region (Rize, Turkey) because of its geographical and climatic conditions. Therefore, we examined the beneficial effects of the extract of these fruits in the present study. The present study investigated the antioxidant effects of whortleberry against cisplatin-induced nephrotoxicity in rats by performing biochemical, histopathological, and immunohistochemical analyses.

## Material and method

Sprague Dawley female rats about 3–5 months and weighing 263.68 ± 8.29 g were procured from Recep Tayyip Erdogan University Animal Care and Research Unit. All animals received humane care according to the criteria outlined in the ‘Guide for the Care and Use of Laboratory Animals’ prepared by the National Academy of Sciences and published by the National Institutes of Health. The study was approved by Recep Tayyip Erdogan University Animal Ethical Committee (ID: 2016/7).

### Study design

Animals care was performed in Recep Tayyip Erdogan University Animal Care and Research Unit. All animals were maintained and fed in a sterile, 55–60% humidity, the temperature of 22 ± 3° C, 12 h light, and 12 h dark experimental animal unit environment. Rats were allowed access to commercially available standard rat chow (Bayramoğlu Feed and Flour Industry Trading Corporation Erzurum, Turkey) and tap water *ad libitum* along the experiment. After sufficient time to comply with the laboratory conditions, 48 experimental animals were divided randomly into six groups (Table 1).

**Table 1.** Experimental study.

Group	Rat numbers
Control	8
Ethanol	8
Whortleberry 100 mg	8
Cisplatin	8
Cisplatin + Whortleberry 100 mg-treated	8
Cisplatin + Whortleberry 200 mg-treated	8

Anesthesia was performed to all experimentals with 50 mg/kg intraperitoneal Ketamine hydrochloride (Ketalar^®^, Eczacibası Parke-Davis, Istanbul, Turkey) and 10 mg/kg intraperitoneal Xylazine HCl (Alfazyne^®^, Alfasan International BV Woerden, Holland). All groups were fed by oral distilled water daily for eight days. Group 1 was determined as a control group (*n* = 8). No drugs were injected except for the drugs used for anesthesia. Group 2 was determined as ethanol control group (*n* = 8). Whortleberry extract dissolved in the ethanol. This group received only intraperitoneal ethanol daily for eight days. Group 3 was determined as whortleberry control group (*n* = 8). This group received only intraperitoneal whortleberry extract 100 mg/kg which was dissolved in ethanol daily for eight days. Group 4 was determined as cisplatin control group (*n* = 8). 16 mg/kg single dose cisplatin (Cisplatin DBL 100 mg/100 mL vial, Orna Ilac, İstanbul) was given intraperitoneally on the 5th day [1,19]. Group 5 was determined as 16 mg/kg cisplatin +100 mg/kg whortleberry group (*n* = 8).

100 mg/kg whortleberry extract intraperitoneally was given daily for eight days and 16 mg/kg single dose cisplatin was given intraperitoneally on the 5th day [13, 20]. Group 6 was determined as 16 mg/kg cisplatin +200 mg/kg whortleberry group (*n* = 8). 200 mg/kg whortleberry extract intraperitoneally was given daily for eight days and 16 mg/kg single dose cisplatin was given intraperitoneally on the 5th day [13,20].

### Preparation of the whortleberry extract

Whortleberry extract (Bilberry [*Vaccinium myrtillus*] Herbal Liquids, Health Aid, England) containing a mix of half distilled water and half ethanol with 330 mg/ml of whortleberry extract was used as a nutritional support product. The product was extra-diluted in sterile conditions with a mix of half distilled water and half ethanol to achieve a whortleberry concentration of 50 mg/ml.

### Biochemical analysis procedure

The kidneys were removed from the abdominal cavities of the rats. Then, they were washed in ice-cold phosphate-buffered saline (PBS). All kidney samples were weighed and homogenized in PBS (pH 7.4) for five minutes. The ratio of tissue weight to homogenization buffer was 1:10. The homogenates were centrifuged at 2717 × *g* for 20 min at 4 °C. Part of the resulting supernatants was used for the determination of Total Antioxidant Status (TAS) and Total Oksidant Status (TOS) by autoanalyzer Architect c16000 Autoanalyzer, Abbott Diagnostics, Waltham, MA [21,22].

### Histopathological analysis procedure

The kidney was fixed in 10% neutral formaldehyde. After the fixation, specimens were dehydrated in an ascending series of alcohol, cleared in xylene, and embedded in paraffin by routine laboratory methods. Preparations of sections were stained with H&E and analyzed by a two blinded histologist to the study groups under a light microscope (DM6200, Leica, Wetzlar, Germany). The findings observed in the photos were taken with the Olympus DP20 camera [22].

### Immunohistochemistry (IHC) analysis procedure

Following steps were performed for Caspase 3 staining: the sections were deparaffinized and treated with proteinase K solution (20 μg/mL in PBS, Abcam, UK), washed in distilled water, and immersed in 3% hydrogen peroxide. After several washes with PBS (10×, pH 6.0, ab128983, Abcam, UK), the sections were immersed in an equilibration buffer. Sections were incubated with Caspase-3 (1:200, Abcam Rabbit polyclonal to active Caspase-3, Abcam, UK). After several washes, all sections were incubated in secondary antibody [Mouse and Rabbit Specific HRP/DAB (ABC) Detection IHC kit (ab64218, Abcam, UK)], and then all sections were incubated in anti-digoxigenin-peroxidase. The reaction was revealed with 0.06% 3, 3-diaminobenzidine tetrahydrochloride (Sigma Chemical, St. Louis, MO) in PBS and the sections were counterstained with Harris hematoxylin [22].

### Quantitative analysis

**Figure 1.**
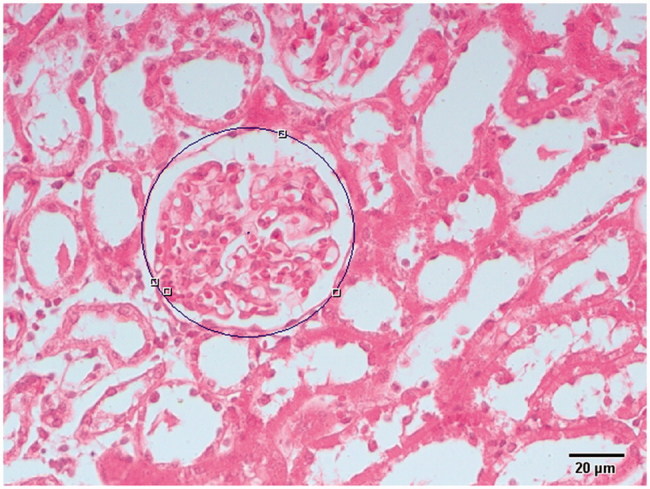
Proximal and distal tubules area (μm2) were calculated using The Olympus DP2-BSW (Ver.2.1 to Ver.2.2, Build 6212, Tokyo, Japan) software system.

In this study, the proximal and distal tubules area measurement (μm^2^) were calculated using The Olympus DP2-BSW (Ver.2.1 to Ver.2.2, Build 6212, Tokyo, Japan) software system was used. This system consists of a camera (Olympus DP20) attached to a light microscope (Leica DM6200-Germany) and a computer with a software system. Hematoxylin & Eosine (H&E)-stained sections were put on the microscope tray, and their sectional boundaries were determined using this program. After determining the area, frames separated from each other were determined by pathologist random [22] (Figure 1).

### Stereological analysis

The mean Caspase 3 positive cell numerical density was calculated using the fractional method, one of the stereological methods with an unbiased counting frame. The Stereo Investigator (MicroBrightField 9.0, Colchester, VT, CA) software system was used. This system consists of a camera attached to a light microscope, a motorized system that carries a microscope tray, and a computer with a software system. IHC-Caspase 3 stained sections were put on the microscope tray, and their sectional boundaries were determined using this program. After determining the area, frames separated from each other were determined by systematic random sampling of the sections, according to the rules of space fragmentation with the step interval of the *x-* and *y*-axis. Then, in 20 different selected areas, of all groups were measured following the method described by Mercantepe [22,23].

### Statistical analysis

Data were statistically analyzed using SPSS Statistics 20.0 software (IBM Inc., Chicago, IL). Biochemical statistical analysis was performed as a mean ± standard deviation, and analyses were performed using differences between the groups were tested using one-way analysis of variance (ANOVA) followed by a Tukey HSD test, and a *p* value <.05 was selected as significant. Statistical analysis of the proximal and distal tubules area measurement area (μm^2^) and Caspase 3 positive cell numarical density were expressed as mean ± standard deviation, and analyses were performed using differences between the groups were tested using one-way analysis of variance (ANOVA) followed by a Duncan test, the numerical data of groups were analyzed (*p* value < .05 was selected as significant).

## Results

### Biochemical results

There was no statistically difference between control, ethanol and whortleberry control groups (*p* > .05). Increased TOS levels and decreased TAS levels were only observed in cisplatin group (*p* < .05). Although a single dose of cisplatin decreased TAS and increased TOS levels, with the treatment of whortleberry extract and cisplatin combination increased TAS levels and decreased TOS levels (*p* < .05). There was no statistically significant difference between 100 mg and 200 mg whortleberry extract (*p* > .05) (Table 2).

**Table 2.** Biochemical data.

Group	Kidney	
	TAS	TOS
Control	9.10 ± 0.28^d^^,^^e^^,^^f^	27.09 ± 0.90^c^^,^^d^^,^^e^^,^^f^
Ethanol	8.57 ± 0.52^c^^,^^d^^,^^e^	30.03 ± 0.83^a^^,^^c^^,^^d^^,^^e^^,^^f^
Whortleberry 100 mg control	9.82 ± 0,57^b^^,^^f^	23.27 ± 0.70^a^^,^^b^^,^^f^
Cisplatin	7.86 ± 0.61^a^^,^^c^^,^^d^^,^^e^	33.02 ± 0.70^a^^,^^b^^,^^c^^,^^d^^,^^e^
Cisplatin + whortleberry 100 mg	9.91 ± 0.33^a^^,^^b^^,^^f^	23.78 ± 0.76^a^^,^^b^^,^^f^
Cisplatin + whortleberry 200 mg	10.43 ± 0.41^a^^,^^b^^,^^f^	22.46 ± 0.99^a^^,^^b^^,^^f^

a *p* < .05 versus to control group.

b *p* < .05 versus to ethanol group.

c *p* < .05 versus to whortleberry 100 mg control group.

d *p* < .05 versus to cisplatin + whortleberry 100 mg group.

e *p* < .05 versus to cisplatin + whortleberry 200 mg group.

f *p* < .05 versus to cisplatin group.

### Histopathological results

#### Light microscopy results

**Figure 2.**
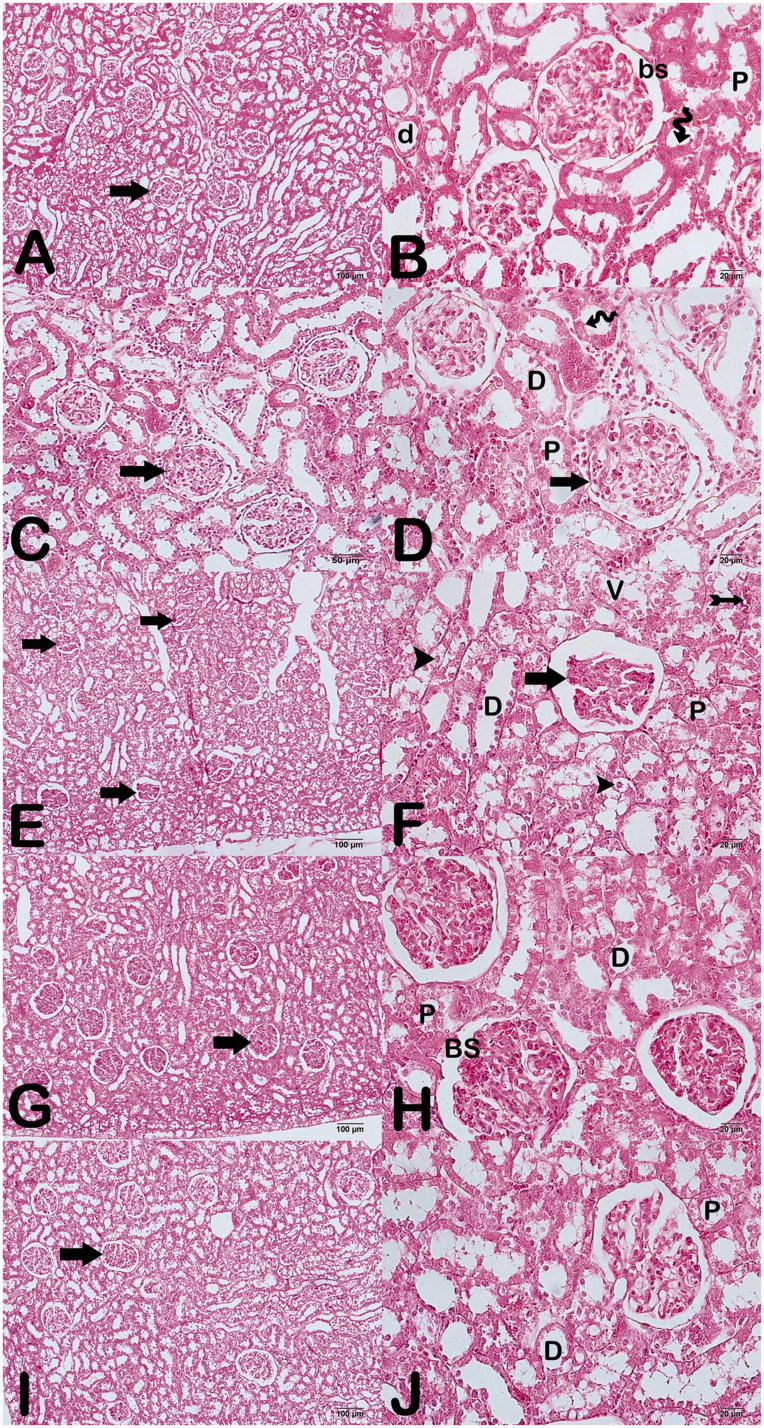
Light microscopic photographs from kidney tissue sections stained with H&E.

Renal tissues sections from control group showed regular Bowman’s capsules with normal renal tissue histological structural features (Figure 2(A)). Ethanol and whortleberry 100 mg groups had normal tubules and Bowman’s capsules were regularly limited. Besides no pathology was monitored (Figure 2(B–D)). However, dilatation, vacuolization and loss of the tubular epithelial cells in the distal and proximal tubules, glomerular degeneration and edema were determined in kidney tissue from cisplatin group (Figure 2(E,F)). In addition, in the remaining tubular epithelium, the connection between the epithelial cell and basement membrane with dense chromatin containing atypical nuclei was lost. In the cisplatin + whortleberry 100 mg-treated group, the Bowman’s capsules and proximal and distal tubules were regular, and no pathology was observed (Figure 2(G,H)). In addition, glomerular degeneration was decreased than cisplatin group. Otherwise, there was vascular congestion in the peritubular spaces (Figure 2(G,H)). Cisplatin + whortleberry 200 mg-treated group, in which the the Bowman’s capsules and proximal and distal tubules were regularly similar to the cisplatin + whortleberry 100 mg-treated group (Figure 2(I,J)).

### Immunohistochemical (IHC) results

**Figure 3.**
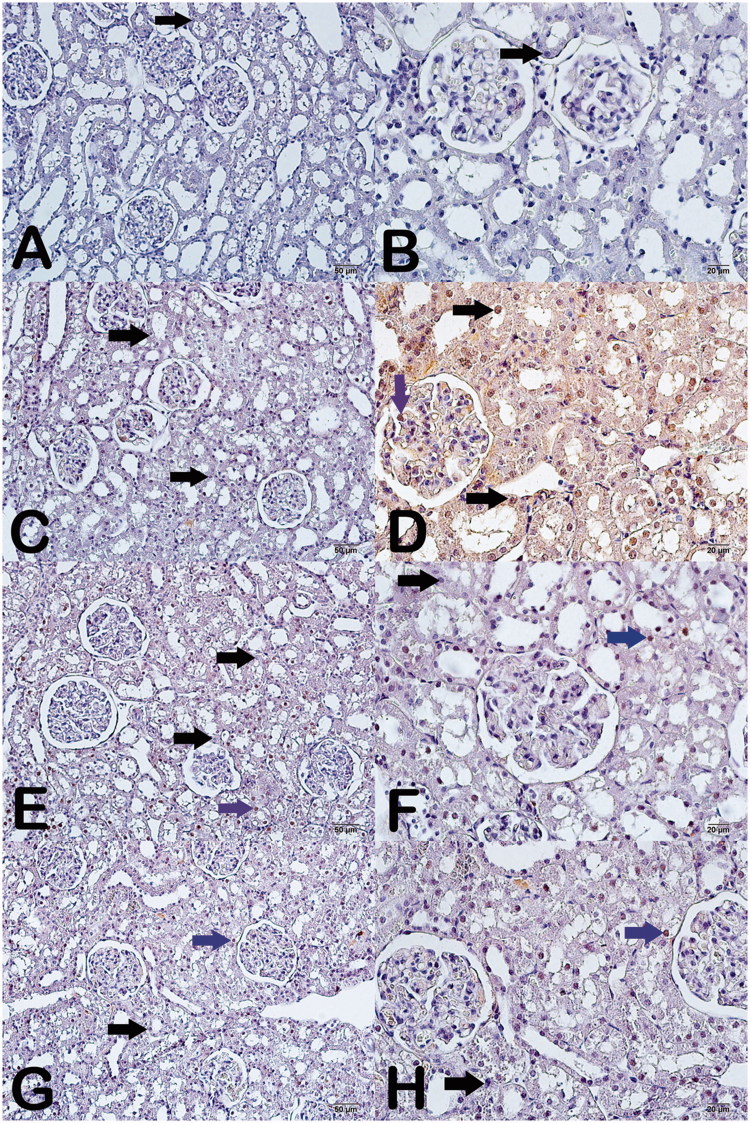
Light microscope image of testicular tissue by light microscopy. Caspase-3 staining.

The results also revealed larger Caspase-3 negative cell numerical density of the proximal and distal tubular epithelial cell of the cisplatin group compared to the control group (*p* < .05) (Figure 3(A,D)) (Table 5). Cisplatin + whortleberry 100 mg-treated group and cisplatin + whortleberry 200 mg-treated group showed less Caspase-3-negative cell numerical density than the cisplatin group (*p* < .05) (Figure 3(A, E–H)) (Table 5). Caspase-3-negative cell numerical density the same markers in cisplatin + whortleberry 100 mg-treated group sections was similar to those of the cisplatin + whortleberry 200 mg-treated group (*p* > .05) (Figure 3(E–H)) (Table 5). The results also revealed Caspase-3-negative cell numerical density of the epithelial cell area of the ethanol and whortleberry 100 mg groups similarly compared to the control group (*p* > .05) (Figure 3(B,E,F)) (Table 5).

### Statistical results

The results also revealed larger proximal and distal tubules area measurement of the cisplatin group compared to the control group (*p* < .05). Cisplatin + whortleberry 100 mg-treated and cisplatin + whortleberry 200 mg-treated group showed decreased proximal and distal tubules area than the cisplatin group (*p* < .05). Proximal and distal tubules area for the same markers in cisplatin + whortleberry 100 mg-treated group sections was similar to those of the cisplatin + whortleberry 200 mg treated-group. The results also revealed proximal and distal tubules area measurement of the epithelial cell area of the ethanol and whortleberry 100 mg groups similarly compared to the control group (*p* > .05) (Tables 3 and 4).

**Table 3.** Proximal tubules area measurement (µm^2^) data.

Group	Proximal tubules area measurement (Mean ± Standard deviation)
Control	2646.42 ± 292.15^a^^,^^c^
Ethanol	2618.13 ± 421.87^c^^,^^d^
Whortleberry 100 mg	3265.65 ± 887.97^a^^,^^c^
Cisplatin	5839.64 ± 1269.50^b^
Cisplatin + whortleberry 100 mg	3541.78 ± 2386.53^a^^,^^c^^,^^d^
Cisplatin + whortleberry 200 mg	2811.12 ± 483.26^a^^,^^c^^,^^e^

a *p* > .05 versus to control group.

b *p* < .05 versus to control group.

c *p* < .05 versus to cisplatin group.

d *p* > .05 versus to cisplatin + whortleberry 200 mg group.

e *p* > .05 versus to cisplatin + whortleberry 100 mg group.

**Table 4.** Distal tubules area measurement (µm^2^) data.

Group	Distal tubules area measurement (Mean ± Standard deviation)
Control	3602.18 ± 271.87^a^^,^^c^
Ethanol	3890.18 ± 341.13^c^^,^^d^
Whortleberry 100 mg	3844.42 ± 360.90^a^^,^^c^
Cisplatin	5619.00 ± 693.77^b^
Cisplatin + whortleberry 100 mg	3014.32 ± 588.33^a^^,^^c^^,^^d^
Cisplatin + whortleberry 200 mg	3308.31 ± 582.82^a^^,^^c^^,^^e^

a *p* > .05 versus to control group.

b *p* < .05 versus to control group.

c *p* < .05 versus to cisplatin group.

d *p* > .05 versus to cisplatin + whortleberry 200 mg group.

e *p* > .05 versus to cisplatin + whortleberry 100 mg group.

**Table 5.** Caspase-3 positive numerical density measurement (mm^3^) data.

Group	Caspase-3 positive numerical density (Mean ± Standard deviation)
Control	4.33 ± 1.03^c^^,d^
Ethanol	4.67 ± 2.16^a^^,c,d^
Whortleberry 100 mg	4.83 ± 2.93^a^^,c^
Cisplatin	44.17 ± 17.53^b^
Cisplatin + whortleberry 100 mg	13.00 ± 3.37^a^^,c,d^
Cisplatin + whortleberry 200 mg	11.25 ± 4.89^a^^,c,e^

a *p* > .05 versus to control group.

b *p* < .05 versus to control group.

c *p* < .05 versus to cisplatin group.

d *p* > .05 versus to cisplatin + whortleberry 200 mg group.

e *p* > .05 versus to cisplatin + whortleberry 100 mg group.

## Discussion

Most of the scientific studies were investigated the effects of antioxidants on nephrotoxicity. On the other hand, our study is the first experimental showing to the antioxidant effect of the whortleberry on cisplatin-induced nephrotoxicity in the literature. We used the cisplatin as an oxidant agent to obtain nephrotoxicity. In addition, whortleberry extract was performed as an antioxidant agent to reduce oxidative process. Although whortleberry extract combined with cisplatin, we observed that application of both 100 mg and 200 mg whortleberry extracts decreased the TOS levels and increased TAS levels, as well. Any significant statistical difference was not observed between 100 mg and 200 mg whortleberry treatment. As a result of the biochemical procedure, we revealed the protective effect of whortleberry extract against cisplatin-induced nephrotoxicity.

Although cisplatin is widely used a broad-spectrum chemotherapeutic agent in standard treatments of many solid tumors, it has serious adverse effects on the various system [24]. Most common observed toxicities of cisplatin are nephrotoxicity, gastrotoxicity, myelosuppression, allergic reactions, hepatotoxicity, ototoxicity, and genotoxicity [5,25]. Miller et al. presented that while cisplatin is being used, various clinical disorders such as acute kidney injury, hypomagnesemia, Fanconi-like syndrome, distal renal tubular acidosis, hypocalcemia, renal salt wasting, and hyperuricemia could be occurred [26]. Histopathological findings like interstitial congestion, an acute tubular injury which points out the nephrotoxicity was shown in an experimental study [27]. Yao reported in his review that from the beginning of the treatment just a few days later, approximately 33% of patients who performed high-dose cisplatin have a severe renal injury [28]. Cisplatin can quickly filter through the glomerular basal membrane due to low molecular weight and accumulates in the proximal tubular inner medulla and outer cortices. Because of the gradient difference, proximal tubules are more affected than distal and collecting tubules [29,30]. Oh and his friends presented that the tubular cell death was a main underlying histopathological feature of nephrotoxicity in their experimental study [24]. Various and complicated mechanisms including oxidative stress, DNA adducts, inflammation, mitochondrial dysfunction, apoptosis, and direct cytotoxicity are responsible for the nephrotoxicity of cisplatin [28,31]. Similar results were found histopathologically in our study. There was widespread loss of the tubular epithelial cells of the proximal and distal tubules in cisplatin group. In addition, glomerular degeneration increased, as well.

In the literature, it is controversial the effect of gender differences on cisplatin-induced nephrotoxicity. Although several studies showed protective effect of estradiol on cisplatin-induced nephrotoxicity [32,33], some studies reported no differences between gender [34,35]. In another study observed increased toxicity associated with high level of estradiol [32]. In present study, nephrotoxicity was observed in control group with cisplatin administration. Hence, we considered that in this experimental study estradiol did not protect kidney from cisplatin-induced nephrotoxicity.

Whortleberry which is a member of bilberry (*V. myrtillus*) family is a native fruit, growing in Rize where states North-east of Turkey. It could be widespread found in the herbaceous layer located plateaues around the mountainous terrain. It prevents various biomedical activities such as inflammatory process, age-related oxidative stress, various degenerative disorders [20]. This protective effect of whortleberry may occur via its high content of anthocyanin pigments. Anthocyanin is a soluble pigment which is consisted naturally and belongs to flavylium cation structure [13]. Thanks to anthocyanins, bilberries have commonly biological effects such as anti-inflammatory, anticarcinogenic, antioxidant, and remaining normal physiological functions [14,18]. Antioxidant effect of bilberry was obtained by Bao et al. in their mice model [13]. It was shown that bilberry could distribute in many organs such as liver, kidney, testes, and lung [36]. Ali et al. revealed that owing to its dual effect of both antioxidant activity and renal distribution, anthocyanin protects kidney against inflammation and fibrosis [37]. It is well known that the use of high dose cisplatin may be one of the pathways leading to nephrotoxicity by activating the Caspase pathway resulting in apoptosis [38,39]. In this present study, we revealed that performing both whortleberry extract 100 mg and 200 mg prevent the deterioration of the Bowman’s capsules, proximal and distal’s tubules epithelial cells from the toxic effects of cisplatin, in view of the histopathologically. But only increased Caspase-3 activity observed with cisplatin in proximal tubule, distal tubule and podocyte cells not also decreased Caspase-3 activity was observed in other groups. These results from our study support that the whortleberry has an antioxidant content.

## Conclusions

In recent times, exposure to oxidative radicals is increasing steadily because of an increase in industrialization. Moreover, the use of oxidants in foods consumed daily or as drugs has prompted several researchers to identify substances with antioxidant properties. The results of the present study showed the antioxidant effects of whortleberries, which are grown abundantly, can be obtained easily and are inexpensive, against cisplatin-induced nephrotoxicity. Therefore, we believe that whortleberries should be included in the daily diet of patients showing oxidative stress to induce antioxidant effects. Moreover, additional large-scale, population-based randomized controlled trials should be performed to determine the antioxidant effects of whortleberries in humans.
